# The Distance Between: An Algorithmic Approach to Comparing Stochastic Models to Time-Series Data

**DOI:** 10.1007/s11538-024-01331-y

**Published:** 2024-07-26

**Authors:** Brock D. Sherlock, Marko A. A. Boon, Maria Vlasiou, Adelle C. F. Coster

**Affiliations:** 1 School of Mathematics and Statistics, University of New South Wales, Sydney, NSW 2052 Australia; 2 Department of Mathematics and Computer Science, Eindhoven University of Technology, P.O. Box 513, 5600 MB Eindhoven, The Netherlands; 3 Faculty of Electrical Engineering, Mathematics and Computer Science, University of Twente, P.O. Box 217, 7500 AE Enschede, The Netherlands

**Keywords:** Distance metrics, Time-series data, Multiple experiments, Distance between evolving distributions

## Abstract

While mean-field models of cellular operations have identified dominant processes at the macroscopic scale, stochastic models may provide further insight into mechanisms at the molecular scale. In order to identify plausible stochastic models, quantitative comparisons between the models and the experimental data are required. The data for these systems have small sample sizes and time-evolving distributions. The aim of this study is to identify appropriate distance metrics for the quantitative comparison of stochastic model outputs and time-evolving stochastic measurements of a system. We identify distance metrics with features suitable for driving parameter inference, model comparison, and model validation, constrained by data from multiple experimental protocols. In this study, stochastic model outputs are compared to synthetic data across three scales: that of the data at the points the system is sampled during the time course of each type of experiment; a combined distance across the time course of each experiment; and a combined distance across all the experiments. Two broad categories of comparators at each point were considered, based on the empirical cumulative distribution function (ECDF) of the data and of the model outputs: discrete based measures such as the Kolmogorov–Smirnov distance, and integrated measures such as the Wasserstein-1 distance between the ECDFs. It was found that the discrete based measures were highly sensitive to parameter changes near the synthetic data parameters, but were largely insensitive otherwise, whereas the integrated distances had smoother transitions as the parameters approached the true values. The integrated measures were also found to be robust to noise added to the synthetic data, replicating experimental error. The characteristics of the identified distances provides the basis for the design of an algorithm suitable for fitting stochastic models to real world stochastic data.

## Introduction

Data from biological experiments is often in the form of time-series data, with multiple samples at each time point. Mean-field models are often used to model this data, and many techniques have been developed to constrain the parameter values to data (see for instance, Press 2007; Gelman et al. 1995). Mean-field models however do not always provide insights into the mechanisms underlying the observed behaviour. Stochastic models, on the other hand, can capture the inherent variance in biological systems and embody mechanistic behaviour that mean-field models may not.

The current investigation was motivated by a system in which there were experimental datasets obtained from different experimental conditions and protocols, each with sparse samples across time. The associated modelling seeks to explain the observations of the system under the different conditions, use these to constrain the model parameters and infer the points at which different perturbations of the system act. Many biological experiments produce similar datasets in which the data consists of a small number of observations of a dynamical system at set time points. Each observation is a sample of an evolving distribution. As the processes are a dynamical system, each time point is dependent on the previous time points. However, the observations are destructive and so paired data is not available. Thus the data is dependent, but not linked, making it unsuitable for many of the existing methods for fitting stochastic models (Albano et al. 2011; Kravtsova et al. 2023; Lanzante 2021).

The suitability of a mathematical model to explain or predict experimental observations requires a comparison of the model output and the data—a quantification of the distance between them. This underlies parameter inference techniques, such as maximum likelihood or Approximate Bayesian Computation (ABC). One common approach to quantifying the distance for stochastic models takes multiple instances of the model output and creates summary statistics to compare to the summary statistics of the experimental data. This requires however, a choice to be made for both the summary statistics and the distance. It has been found that the choice of summary statistics is crucial to the quality of the inference and a poor choice can lead to a loss of information (Burr and Skurikhin 2013; Schälte and Hasenauer 2023). Summary statistics are also affected by sample size. Indeed, the summary statistics approach can be difficult to interpret and is troublesome for quantitative model comparison (Beaumont et al. 2002). There are methods to compare distributions when the continuous probability distribution can be estimated (Schmid and Trede 1995; Belov and Armstrong 2011; Zhao et al. 2022; Iannario et al. 2024), however, the performance of probability density estimation is again reliant on sample size (Farmer and Jacobs 2018).

In the case of stochastic differential equation models, fitting methods have used the analytical form of the equations to make comparisons to analytical moments, or make assumptions on the distributions (Kügler 2012; Albano et al. 2011; Craigmile et al. 2023). However, in many cases the underlying distributions of the data are unknown and there are no analytic forms to exploit.

This investigation focuses on the assessment of distance measures combining multiple different, sparsely sampled, time-evolving experiments. Ideally the overall distance should smoothly decrease as the model parameters are adjusted to better represent the observations. For small sample sizes, summary statistics are not able to be robustly estimated and hence we were motivated to focus on non-parametric methods to develop a distance measure, to explore whether our hypothetical model was plausible.

The algorithm proposed in this paper calculates distances directly on the model outputs and observed data, avoiding both the possible effects of assumptions about distributions and the choice of summary statistics, particularly important in this case of sparse data. A similar approach was employed in Bernton et al. (2019) where a single paired time series data set was compared using the Wasserstein distance to inform ABC parameter inference. In the algorithm proposed in this study, the distance is constructed from quantitative non-parametric comparisons at each data point, accumulating these across each time course, and then across each experimental protocol to obtain an overall quantification of the distance between the experimental datasets and the model outputs for a given set of parameters.

For each experiment, the distribution of the data and the model outputs needs to be compared at each sample time. Standard methods for distribution comparisons are designed to determine whether the two samples are drawn from the same distribution or not (Goldman and Kaplan 2018; Kiefer 1959; Anderson and Darling 1952; Pettitt 1976). To enable parameter inference, however, the distance measure needs to not only classify whether the distributions are statistically identical at the different points in each of the experiments, but also quantify the difference in a manner that enables a directed search of the parameter space. This is a particularly desirable feature for computationally expensive models. Inference methods, such as ABC, require some measure of ‘closeness’ between a model output and the data to determine whether one parameter set is better than another.

Here, a hypothetical model is used as a framework to both create synthetic data that mimic sparsely sampled biological experiments and then test the algorithm quantifying the distance between the stochastic model outputs and the distributions of the corresponding experimental data as time evolves. Synthetic data, created using instances of the model with known structure and parameter values allows direct comparisons of other variants of the model to this “ground truth”. Experimental data often includes experimental errors. In other words, one may assume that the data observed is not from the underlying “true” distribution, but rather from the distribution plus some measurement error. To explore these possible effects we add additional noise to the synthetic data to create noisy synthetic data. These noisy synthetic data sets are used to explore the efficacy and characteristics of the distance measures in the presence of measurement error and show the robustness of the distances to the additional noise under re-sampling.

**Fig. 1:**
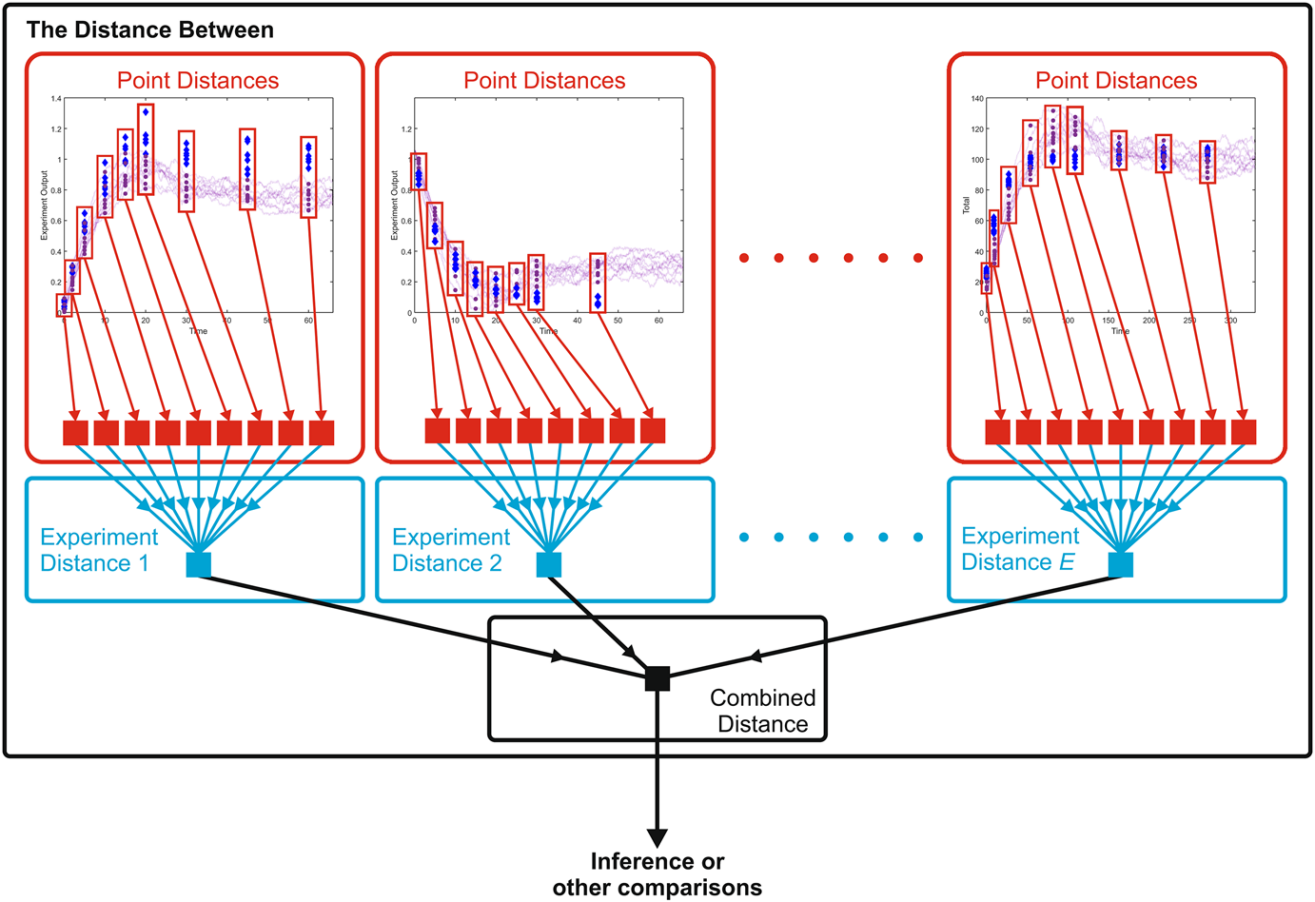
The algorithm to produce a hierarchical distance measure over three scales: individual time points, across a time course of a single experiment, and a combined distance over all *E* experiments. This combined distance can then be used for parameter inference, model or other comparisons (Color figure online)

Data is utilised from several unique experiments, and the algorithm builds a hierarchical distance over three different scales: at individual time points during each type of experiment; an aggregated experiment distance across the time-course of each individual experiment; and an overall distance across all the experiments considered, Fig. 1. As the underlying parameters of the synthetic data are known, the distances can be explored as the parameter values are perturbed away from those of the synthetic data. Desirable features of the distance for parameter inference include a smooth descent to a minimum value in the parameter space when the parameter values approach the “true” values, insensitivity to sampling, and robustness to noise.

The biological system used as an exemplar for the algorithm in this study is the translocation of glucose transport protein 4 (GLUT4) in mammalian adipocytes in response to the application of insulin. GLUT4 is the main facilitative glucose transporter in these cells, allowing the dynamic control of glucose uptake. GLUT4 is membrane-embedded protein and is continually recycled to and from the outer cell plasma membrane. The presence of insulin stimulates GLUT4 translocation from the cell interior to the plasma membrane. Mean-field models have previously been utilised to identify dominant processes at the macroscale (Brewer et al. 2016a, b, 2014; Coster et al. 2004; Fazakerley et al. 2010; Govers et al. 2004; Holman et al. 1994). However, at the microscale, the system is inherently stochastic.

To capture the stochasticity of the biological system, we hypothesised a queuing network model. In order to see whether this new model of the GLUT4 translocation process is appropriate we required a quantitative comparison for the sparsely sampled datasets for the experiments, and hence the current study. The inference of the optimal parameters of the model for the actual experiments and an assessment of the efficacy of the model as a description of the biological processes is the subject of a subsequent study.

The stochastic model to be considered in this case was a closed queuing network. A queue is a stochastic model that takes the timing of arrival of a customer at a service station and probabilistically determines their departure time based on the state of the system at the arrival time and the service protocol for dealing with arrivals, such as first-in-first-out. Queuing Networks are stochastic models where customers are routed through a network of service stations. Upon arrival at the station the customer either waits for service in the queue if the server is busy or is processed with some stochastically drawn service time. The customer is then routed to the next service station in the network or exits the system. In a closed queuing network the customers neither exit nor enter the network but continuously cycle through the service stations. Further information on queuing can be found in, for instance, Asmussen (2003). Queuing models have been used in many applications including telecommunication networks (Beneš 1957), internet and computer networks (Baccelli et al. 2009), and traffic problems (Amini et al. 2016). More recently, queues have been used in biological systems such as inhalation toxicology (Wu 1998), glycolysis (Clement et al. 2020), and Krebs cycle modelling (Kloska et al. 2021).

Most work in parameter inference for queuing systems focuses on single queues, of varying types (Amini et al. 2016; Abramov 2011; Armero 1994; Armero and Bayarri 1994; Basawa et al. 1996; Basawa and Prabhu 1988; Beneš 1957; Bhat and Rao 1987; Bingham and Pitts 1999; Clarke 1957; Ross et al. 2007; Wang et al. 2006; Wolff 1965), and some work on open networks (Alouf et al. 2001; Baccelli et al. 2009; Mandelbaum and Zeltyn 1998; Rohrscheidt 2017; Thiruvaiyaru et al. 1991). The classical approach to queuing parameter estimation requires continuous observation over a fixed (possibly predetermined) time period (Clarke 1957; Armero 1994; Thiruvaiyaru et al. 1991; Beneš 1957; Basawa and Prabhu 1988; Bhat and Rao 1987; Wolff 1965). In our motivating case, continual observation of the biological systems of interest is impossible as all measurements of the system are destructive. More recent techniques use ‘active probing’, the ability to probe the system at user specified times, rather than requiring continual observation (Baccelli et al. 2009). However, the probing used assumes complete visibility of the system, i.e., the ability to probe any location of the network. This level of visibility is not permitted in the cellular observations for our system, where observations are restricted to only those from the cell surface.

The proposed multi-scale approach to determining distances between stochastic models and stochastic time-series data provides a basis for quantitative comparison of stochastic models with data. This approach also allows data from various sources to constrain the model parameters, which can aid researchers in fields where experimental sample sizes are small but where data can be gathered from multiple sources. The robustness of distances to experimental noise and sampling improves confidence when fitting parameters when the sample size is small and experimental noise present.

## Methods

### Biological Background

The translocation of GLUT4 in response to insulin has been observed using immunofluorescence experiments (Govers et al. 2004, 2008; Brewer et al. 2011, 2014, 2016a, b) in which GLUT4 molecules carrying fluorescent tags were tracked under different conditions. In such experiments, the amount of GLUT4 at the plasma membrane (i.e., at the cell surface), or that which has transited to the plasma membrane, was assayed. The experimental data consists of a small number of repeated measurements at distinct times over the time course of each experiment. These are bulk measurements and the results are the average over many thousands of cells. In addition, each measurement is destructive, which implies that measurements over time are of the same cell type but not of the exact same cells.

Two experimental protocols are considered for this study. The *transition experiment* tracks the amount of GLUT4 present at the plasma membrane as a function of time (Brewer et al. 2014) (see Fig. 3 for an example of experimental data). This experiment begins in the basal (zero insulin) steady state. At time zero, a level of insulin is applied, perturbing the system towards a new steady state (the insulin steady state). The experiments tracked the surface level of GLUT4 at distinct sample times up to 60 minutes. Note that, due to practical considerations in the real experiments, the initial insulin level is always less than or equal to the final concentration of insulin, i.e., insulin can not be removed from the system for these experiments.

The *uptake experiment* tracks the cumulative amount of GLUT4 that has visited the plasma membrane as a function of time from the experiment onset in the presence of a fixed amount of insulin (Brewer et al. 2014) (see Fig. 3). As the cells are in steady state, the total amount of GLUT4 at the surface remains the same; however, the cumulative amount of GLUT4 that has transited through the cell plasma membrane is an increasing function due to recycling. This cumulative amount is tracked by labelling the GLUT4 molecules when they visit the surface and quantifying the total amount of labelled GLUT4 at the experimental measurement times. In this study, we consider two cases of the uptake experiment: the basal case (zero insulin) and the ‘insulin’ case (maximum insulin, or 100 nM).

The experimental measures were normalised to the mean of the values at the final time point under maximum insulin stimulation, i.e., all uptake measurements are normalised to the steady state of the uptake experiment in 100 nM insulin and all transition measurements (also perturbed with 100 nM insulin) are normalised to the 60 minute values, by which time it is assumed the system has achieved steady state.

As mentioned above, the current investigation was motivated by our wish to test hypotheses about the mechanisms controlling the insulin response in this system. Here we use a hypothetical model as a test bed to test the efficacy of different comparison methods between stochastic models and data.

Consider a closed recycling network, comprised of various structures in the cell at which GLUT4 can be located. These include the outer cell plasma membrane (where the GLUT4 molecules can facilitate glucose transport into the cell), the internal endosome membranes, and vesicles, which are small, mobile spheres of membrane, which can be actively transported down microtubules in the cell. These microtubules form a network of tracks within the cell, connecting regions near the inner endosomal membrane structures to the underside of the outer plasma membrane. Via coupling to a molecular motor, vesicles are transported along the length of a microtubule from the endosomal stores to the plasma membrane (Semiz 2003). Here it is assumed that each molecular motor transports a single vesicle at a time with a unique speed distributed about some average.

The microtubules have finite length (about $10\, \mu$m, Semiz 2003) so the model below assumes that some finite number of vesicles are able to occupy the microtubule at any time, proportional to the size of the vesicles. It is also assumed that vesicles attach and detach at the ends of a microtubule, and do not overtake other vesicles that are already attached to the microtubule.

**Fig. 2:**
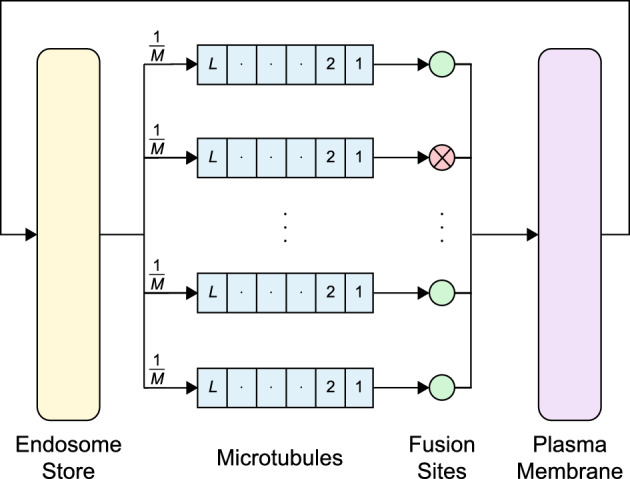
Schematic of the closed queuing network. *N* vesicles are routed cyclically through the endosome store, the *M* microtubules, the *M* associated fusion sites and plasma membrane. Arrows denote the direction of travel through the system. Split paths have the probability of a particular branch being chosen shown, i.e., a microtubule branch is chosen with probability $\frac{1}{M}$. The microtubules queues have capacity *L*, and the fusion sites are either active or inactive set by $p_B$ and $p_I$ (green for active, red with cross for inactive) (Color figure online)

At the end of the microtubules the vesicles are delivered to fusion sites, at which, SNARE proteins facilitate the fusion or merging of the vesicle membrane with the plasma membrane, transferring the associated GLUT4 (see for instance Stöckli et al. 2011). In this model the activity of the fusion sites is insulin-dependent, with each individual site predetermined to be either active or inactive depending on the insulin present in the system. The greater the insulin dose, the greater the number of active sites. The recycling pathway of GLUT4 in the model is shown in Fig. 2.

### Mathematical Model

The particular model implemented here for the recycling of GLUT4 is a closed queuing network with four stations: the endosome store, the microtubules, the fusion sites and the plasma membrane; see Fig. 2. A closed queuing network is one in which the customer population is constant and is routed internally within the system; there are no external arrivals or departures. The *N* customers in this network are the vesicles. Although vesicles are physically destroyed and reformed at the plasma membrane in the biological system, we assume that the number of vesicles in the biological system remains constant, and quantify the amount of GLUT4 at the plasma membrane in units of vesicles. The vesicles in the system cycle through the stations in an ordered manner: from endosomes to microtubules, to fusion sites, to the plasma membrane, and then back to the endosomes. The service time at each station is exponentially distributed and the switch times between end of service at one station and entry to the queue of the next are taken to be zero, i.e., movement to the next station is incorporated in the previous service time.

We assume the endosomes have sufficient capacity to hold all vesicles in the system at any given time. Thus, the endosome station is an infinite-server queue. Biologically, the service times represent the time needed for a vesicle to exit the endosomes and arrive at a microtubule if unimpeded. The service times are exponentially distributed with rate $\mu _S$. Vesicles (customers), at completion of service, attempt to move to the next station, i.e., the microtubules.

Physically, microtubules have finite vesicle capacity. We assume that only a finite number of vesicles, *L*, can be physically attached along their length. The microtubule station is modelled as *M* parallel multi-server queues, each with *L* servers and zero buffer. Each of the *M* queues maintains first in first out (FIFO) ordering (no overtaking), and it is assumed that the vesicles only attach and detach at the endpoints of the microtubule. Whilst attached, they can only move along the microtubule maintaining FIFO ordering.

Upon arrival at the microtubule station the vesicle chooses a particular queue with probability $\frac{1}{M}$. The next action depends on the capacity of the chosen queue. If the chosen queue is at capacity, i.e., all *L* servers are occupied, the vesicle is blocked and re-enters service at the endosome station (redrawing a new endosome service time). If the vesicle selects a microtubule queue with available servers, it immediately enters service.

Each microtubule is associated with a particular fusion site. The processing of the vesicle in the microtubule queue is dependent on the departure of the previous vesicles, and the capacity of the associated fusion site. If unimpeded, the service time of the vesicle is drawn from an exponential distribution with rate $\mu _M$, representing the unimpeded transit time along the entire length of the microtubule. If the vesicle arrives at the associated fusion server and finds it unavailable, the vesicle is blocked and waits until the fusion server is ready. Any previous vesicles waiting in the microtubule queue block the current vesicle from transiting the entire length of the microtubule. As a queued vesicle departs, each blocked vesicle waiting behind it in the queue moves up one vesicle diameter, due to the FIFO mechanism, filling the place vacated by the previous vesicle. This movement requires an ‘increment time’ sampled from an exponential distribution with rate $\left( L\cdot \mu _M\right)$.

The fusion station comprises *M* parallel single-server queues with zero buffer and exponentially distributed service times with rate $\mu _F$. Vesicles arrive at a fusion site from their associated microtubule, i.e., vesicles leaving microtubule queue *m* are routed to fusion site *m*. The fusion service time encapsulates the time the fusion site needs to grapple the vesicle, process it, and allow it to fuse to the plasma membrane. Each fusion site is either active or inactive in a given instance of the model, fixed by an insulin dependent active probability parameter, $p_B$ in the basal (or no insulin state) and $p_I$ in the presence of insulin. In this model, the state of a fusion site only changes with an insulin change, i.e., a fusion site remains in its current state until a perturbation of the insulin level. If the fusion site is active, arrivals immediately enter service as soon as the site is available. However, if it is inactive then it does not accept any vesicles and blocks the path to the plasma membrane. This also prevents vesicles from departing the associated microtubule. Blocked vesicles in the microtubule are immediately transferred when the fusion site server becomes available.

Upon completion of service at the fusion sites, vesicles are deposited to the plasma membrane, which is modelled as an infinite server queue—it has sufficient capacity to hold all vesicles in the system. The service time at this station encapsulates the time for a vesicle to reform and transit time of the translocation within the cell to return to the endosome store. The service times are exponentially distributed with rate $\mu _{P}$. After service at the plasma membrane vesicles return to the endosome store and begin the cycle again.

### Simulations of Experiments

Three types of biological experiments for each instance of the system were implemented: the transition experiment and two uptake experiments—in basal and in insulin steady state. The parameter values for the system were set, and the activity of each fusion server in the basal and insulin states were pre-determined by the active probabilities $p_B$ and $p_I$, where $p_I>p_B$. This inequality reflects the model hypothesis that insulin activates these sites.

In simulations of the transition experiment, the system is allowed to equilibrate with the activity of the fusion servers set to the basal state. Then, at the start of the experiment, the activity of the fusion servers is switched to their insulin state, modelling the application of insulin. In the model, the GLUT4 level on the cell surface is determined by the queue length of the plasma membrane station. This data was recorded at the same time points as in the biological experiments (Brewer et al. 2014). For the transition experiment the measurement times were $\{0,0.5,1,2,5,10,15,20,25,30,45,60\}$ minutes. Sets of samples were gathered for each parameter set and were then normalised to the average value of the steady state level (which, as in the biological experiments, was deemed to have occurred by the final time point).

The uptake experiment tracks the steady state recycling of GLUT4 by tagging the first appearance of the molecule to the plasma membrane. To simulate this, the model system is allowed to equilibrate with the fusion server activity (basal or insulin, depending on the experiment) and then the output logs the cumulative number of unique vesicles that have visited the plasma membrane as a function of time, the initial value being the steady state level at the plasma membrane. Consistent with the biological experiments (Brewer et al. 2014), samples were made at each of the 12 measurement times $\{0,2,5,10,20,30,60,90,120,180,240,300\}$ minutes. Simulations of the basal and insulin uptake experiments are paired in the sense that both are simulated using the same parameter values other than the fusion server activity (which encodes the absence or presence of insulin). Basal uptake and insulin uptake data are normalised to the average value of the samples at the final time point of the insulin uptake experiment.

Numerical Implementation The discrete event simulation of the model was implemented using Java OpenJDK 19.0.1. To minimise the required run time, the experiments were simulated in sequence within the same simulation run. Each run was initialised with all vesicles in the endosome station and the parameters set to the basal state, i.e., fusion server activity determined by the active probability $p_B$. The simulation then ran for 500 simulation time units to allow the distribution of vesicles at each station to reach the first equilibrium, i.e., the basal steady state. The basal uptake experiment was then initiated. After the uptake experiment, the transition experiment commenced with the system perturbed to the insulin parameters; i.e., fusion server activities determined by the active probability $p_I$. From the onset of the transition experiment, the simulation was allowed to run for 500 time units to allow the system to reach the second steady state (the insulin steady state) before the insulin uptake experiment was initiated. Post-processing and visualisation of the results was implemented in Matlab (Version R2020b, Mathworks 2020).

### Synthetic Data

For this study, synthetic data was created for each of the transition, basal uptake and insulin uptake experiments with seven repeat samples at each time point. The sparsity of this sampling is consistent with the biological data in these experiments (Brewer et al. 2014).

**Fig. 3:**
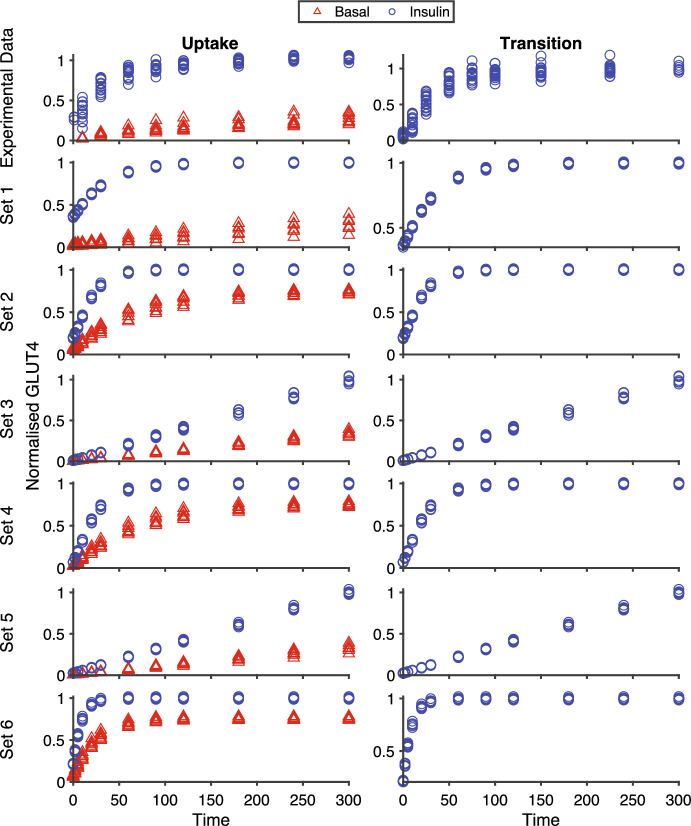
Experimental and Synthetic Data for basal uptake, transition, and insulin uptake experiments. The basal and insulin uptake experiments show the cumulative amount of GLUT4 that has transited to the plasma membrane as a function of time. The transition experiment shows the instantaneous amount of GLUT4 present at the plasma membrane as a function of time after the application of insulin. All amounts are expressed as fractions of the total amount of GLUT4 recycling in the insulin stimulated state. The experimental data from Brewer et al. (2014) is shown in the first row, in which 3T3-L1 adipocytes ($\sim 1000\,\hbox {cells/sample}$) were incubated with 0nM (basal uptake) or 100 nM (insulin uptake) insulin for 45 minutes to reach steady state and then data collected of the amount of GLUT4 that had been labelled at the cell surface for the times indicated. Data points were normalised to the maximum level recycling in 100 nM insulin. The transition experiments incubated the cells with 0 nM insulin for 45 minutes and then the cells were stimulated with 100 nM insulin for the indicated times and then placed on ice and labelled. Synthetic data was simulated using the parameter sets given in Table 1 and was of comprised seven true samples of the simulated system at each time point. These are normalised to the mean of the synthetic data at the final time point. Note the different vertical scales. The time is measured in minutes for the experimental data and arbitrary units for the synthetic data (Color figure online)

Different sets of parameter values were explored, and outputs with qualitative correspondence to the biological data were identified (Fig. 3). Sets were chosen to have qualitative correspondence so the distances can be tested against synthetic data of the same form as observed in biological data. Six parameter sets (Table 1) were used to create synthetic data as true samples of the simulated system at these parameter values. In addition, we created noisy synthetic data that comprised samples of the simulated system with either added Gaussian or uniform noise at each data point to represent different levels of experimental or measurement error.

The model outputs and synthetic data were compared for a range of values around the true parameter values to investigate the behaviour of different distance measures in parameter space (Table 1). For the purposes of this study, four parameter values (the service rates, $\mu _S$, $\mu _F$, and $\mu _P$, and the basal state active probability, $p_B$) were varied. The model network structure (i.e., the number of customers, *N*, the number of microtubules, *M*, and the microtubule capacity, *L*) was fixed. Similarly, the microtubule service rate, $\mu _M$, which can be estimated from knowledge of molecular motor speeds, and the insulin state active probability, and $p_I$, which determines the total number of customers recycling in the insulin state, were taken to be constant.

The parameter ranges given in Table 1 were each divided into 21 equally spaced points. This created a total of 488,187 parameter combinations with 83,349 points around each of the six parameter sets. At each point in parameter space, the queuing model was simulated 100 times and distances for the three experiments determined as functions of the model parameters for each synthetic data set. It was determined that model sample sizes larger than 100 had little to no effect on the distance comparisons.

The distances were investigated as a function of the parameter distance, the scaled Euclidean distance between the model parameters and the synthetic data parameters. As the points surveyed were evenly spaced within the parameter range, the parameter distance is scaled, counting the distance between adjacent points in the parameter space, along any single dimension, as being one distance unit apart.

To investigate the effects of re-sampling on the distances, 1000 sets of synthetic data with 7 samples in each set were created for parameter set 1 (Table 1). This was to ensure the distance metrics are robust to re-sampling and results are not dependent on having a ‘good’ sample. Additionally, noisy synthetic data sets were created by adding Gaussian noise to the synthetic data points at several different levels: 0, 1, 2, 3, 4, 5, 7.5, 10, 12.5, 15, 17.5 and 20 percent. The added noise was drawn randomly from the Gaussian (normal) distribution with mean zero and standard deviation equal to the relative error. That is, 5% noise relative noise indicates drawing a random variable from the distribution ${\mathcal {N}}(0,0.05^2)$. In this setting, the data with noise is then given by

$$\begin{aligned} \text {noisy data} = \text {data} + \left( \text {data} \times \text {random relative error}\right) . \end{aligned}$$

Sampling the noise from the normal distribution allows arbitrarily large values to occur, although with small probability. However approximately 99.7% of values fall within 3 standard deviations of the mean. So, for example, with a standard deviation of 0.05, values as large as 0.15 can occur. For this study, if the sample drawn was such that the noisy data point became negative (a non-physical value) the error was re-sampled. In order to separate the effects of re-sampling and additive noise, the same synthetic data sets were re-used as the basis to construct the different level noisy data sets in the studies. An additional comparison study, where the noise was sampled from a uniform distribution was also undertaken.

## Distances Between Stochastic Models and Data

In order to determine the efficacy of the different distance measures as comparators between stochastic models and data, knowledge of the “ground-truth” parameter values of the data is required. Here, we implement stochastic simulations of a system with known parameters to create synthetic data, both with and without noise. The distance between the stochastic model and the data as a function of the difference in the underlying parameter values can then be determined. This, in some ways, represents the best-case scenario—the model and structure is truly representative of the experimental system from which the data is derived. Investigations of suitable distances and algorithms in this ideal scenario indicate which distance measures may not be amenable to meaningful inference and model selection in the case of less ideal models of reality.

The goal of this study is not to provide an exhaustive review of distance measures. Instead, we aim to identify the types of distance measures that could be used to infer parameters of a system with stochastic data that evolves over time, and identify characteristics of these distances that are useful under different circumstances. Using a combined distance, which accounts for all time points of all experiments, the distance between the synthetic data and model output under a variety of conditions and a range of input parameters can be explored.

Candidate distance measures were selected that could be sensitive to deviations of the parameters in our system. These measures need to compare the distance between samples at a single time point in a particular experiment, aggregate these time point distances into a single distance for an entire time-series of samples, and further combine these across the different experiments that constrain the system.

Where experimental data sets are sparse, multiple independent experiments of the same system can be used to constrain the parameters of the model. The independent experiments can be modelled with common parameters across the different contexts. This may require the creation of multiple instances of a single parameter to suit different conditions and hypotheses, whilst constraining others to be common in all contexts. Model outputs for each experimental context for a given set of parameters can thus be created.

In this study, we focus on non-parametric techniques to compare data consisting of a series of time points, each with a small sample of data values from some distribution. As the data has small sample sizes, we investigated distances between empirical cumulative distribution functions (ECDFs) for which there is no need to impose assumptions about the type of distribution, and thus avoids the introduction of errors through the approximation of mass functions. Moreover, the samples to be compared can have different sizes (and the ECDFs are not computationally expensive to generate).

Consider a set of *E* experiments, $i=1,\dots ,E$, where experiment *i* has time points $t_{ij}$, $j=1,\dots ,\tau _{i}$. Time point $t_{ij}$ has data values $f_{ijk}$, $k=1,\dots ,V_{ij}$, and model outputs $g_{ijm}$, $m=1,\dots ,R_{ij}$, where $V_{ij}$ is the number of experimental repeats of the data, and $R_{ij}$ is the number of samples of the model. The algorithm developed in this study hierarchically compares the distributions of the data and model firstly at each time point within each experiment, then combines these comparisons across each experimental time course, and then combines the distances across the experiments.

### Time Point Distance Comparisons

To compare the correspondence of the data with the model outputs for a given experiment, the distributions are firstly compared at individual time points $t_{ij}$. The distribution comparators are based on ECDFs. The ECDF $P_N(x)$ is defined as

1 $$\begin{aligned} P_N(x) = \frac{1}{N} \sum _{n=1}^N \mathbbm {1}_{x_n \le x}, \end{aligned}$$

where $\mathbbm {1}$ is the indicator function and $x_n$, $n = 1,\dots ,N$, are the data points. Let the ECDFs of the data be $F(x,t_{ij})$ and of the corresponding model outputs be $G(x,t_{ij})$. For distance measures that require a combined sample, the union of the two samples $f_{ijk}$ and $g_{ijm}$ is used.

A disadvantage of using ECDFs for comparing data to model outputs is that the number of instances of the model output must be chosen, which has an effect on the smoothness of the ECDF. The randomness of the sample data means that the distance between the data and different sets of model samples will vary, even if the parameter sets are identical. This variance can be minimised by creating a sufficiently large number of samples of the model output such that the ECDF is reasonably smooth, which in this case was determined to be $R_{ij}=100$ samples for the model outputs for all times $t_{ij}$ and experiments *i*.

In this study, we consider two classes of distance measures between the synthetic data and model ECDFs: those that utilise only discrete sample points on the ECDFs (i.e. the distance between the two ECDFs at each step value), and those that consider the integrated distance between the ECDFs by accounting for all sample points and the spacing between them. The class of discrete distance measures includes the Kolmogorov–Smirnov metric, the Kuiper distance, the Cramer–von Mises distance, and the Anderson Darling distance. The class of integrated distance measures includes the Wasserstein-1, squared area, and the signed area between ECDFs. We found that distances within each of these classes produced similar results; so here we illustrate the characteristics of the discrete distance class with the Kolmogorov–Smirnov metric and the integrated distance class with the Wasserstein-1 distance. The definitions and results for the other metrics are included in Appendices A and B.

**Fig. 4:**
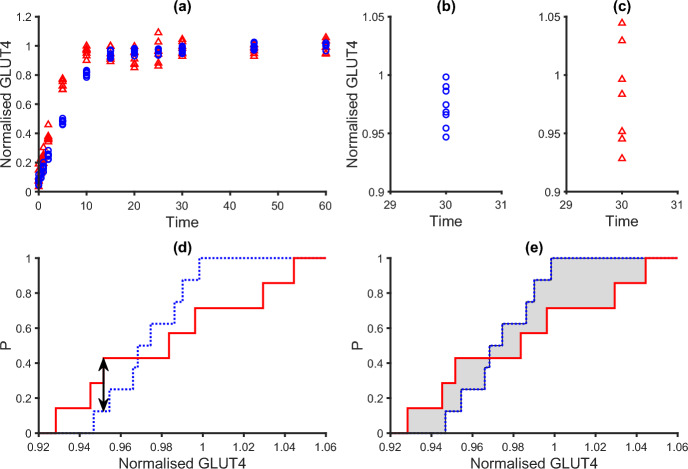
Two example experimental data sets for comparison. Data Set 1 is indicated by blue circles and dotted blue lines. Data Set 2 is indicated by red triangles and solid red lines. **a** The evolution of two data sets with time; **b** Detail of the distribution of Data Set 1 at time $t=30$; **c** Detail of the distribution of Data Set 2 at time $t=30$; **d** The associated ECDFs for the two data sets at $t=30$ indicating the Kolmogorov–Smirnov distance (black arrow); **e** The associated ECDFs for the two data sets at $t=30$ indicating the Wasserstein-1 distance, the area enclosed by the ECDFs, with grey shading (Color figure online)

Consider the ECDFs of the synthetic data $F(x,t_{ij})$ and corresponding model outputs $G(x,t_{ij})$ at time point $t_{ij}$ of experiment *i*. The Kolmogorov–Smirnov Distance, $d_{KS}(t_{ij})$, for this data is defined as the maximum distance between the ECDFs of the synthetic data and corresponding model outputs, i.e.,

2 $$\begin{aligned} d_{KS}(t_{ij}) = \sup _x |F(x,t_{ij}) - G(x,t_{ij}) |, \end{aligned}$$

where *x* is the domain of the combined sample (Darling 1957). The Kolmogorov–Smirnov distance is illustrated in Fig. 4.

A series of integrated distance measures were considered. The area between the ECDFs, or the Wasserstein-1 distance, for the data above is defined as

3 $$\begin{aligned} d_{W_1}(t_{ij}) = \int _{-\infty }^\infty \vert F(x,t_{ij}) - G(x,t_{ij})\vert \,dx, \end{aligned}$$

where *x* is the domain of the combined sample. This study utilises the Wasserstein-1 metric which gives the area between the ECDFs of the two distributions (see Fig. 4). The Wasserstein-1 metric is also known as the Earthmover’s distance, and is commonly used in geology and computer science (see for instance Lipp and Vermeesch 2023). The signed area between the ECDFs and a ‘squared’ L2-area distance were also considered and the definitions and results for these metrics are included in Appendices A and B.

Given a set of data and model samples, there exists a theoretical constraint on the range of possible values for each of these distances. For example, the Kolmogorov–Smirnov distance, $d_{KS}(t_{ij})$, can take values between zero and one. The Kolmogorov–Smirnov distance and other discrete metrics also have a limited number of discrete values. Given two samples, one of size *N* and the other of size *M*, the Kolmogorov–Smirnov distance will return one of only $N \times M$ possible unique values. The Wasserstein-1 distance and other integrated distances can theoretically return any value between 0 and the maximum observed value in the data. As the synthetic data in this study was normalised, the maximum domain of the ECDFs for the experiments, and thus the maximum Wasserstein-1 distance, was constrained to approximately 1.5.

The theoretical bounds for other metric definitions can also be determined. In all cases however, the actual minimum values of the discrete metrics, even if the data and model correspond exactly, are constrained by the sampling levels, i.e., even if the data and the model outputs were drawn from the same distributions, the minimum distances at a given time point are non-zero.

### Experiment Distances

**Fig. 5:**
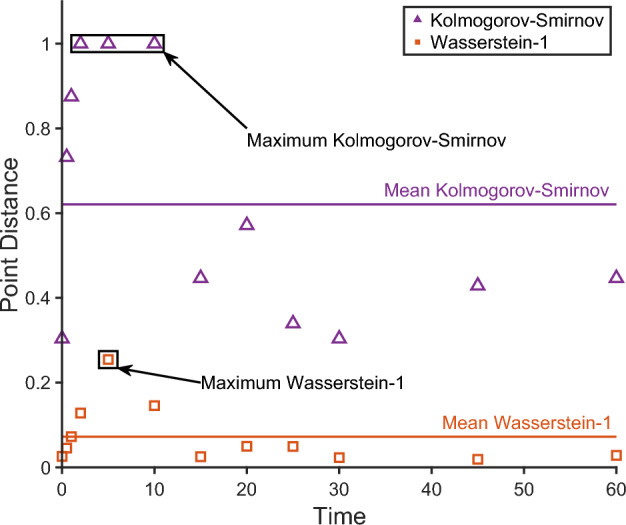
Kolmogorov–Smirnov and Wasserstein-1 point distances as a function of time for the data sets shown in Fig. 4. The resulting maximum and mean experiment distances are shown for each underlying point distance (Color figure online)

The distances above compare two distributions at just a single time point. Figure 5 shows an example of how the Kolmogorov–Smirnov and Wasserstein-1 distances vary with time in the transition experiment for data sets illustrated in Fig. 4. Notice the distances change as a function of time, with points between one and ten time units have much lower correspondence than the early and late time points.

Four methods were explored to combine the vector of point distances, $d(t_{ij})$, $j=1,\dots ,\tau _i$, across the time course to create an experiment distance, $D_i$, between the data and model for experiment *i*. These methods are: the mean,

$$\begin{aligned} {\bar{D}}_i=\frac{1}{\tau _i}\sum _{j=1}^{\tau _i}\left( {w_{ij}d(t_{ij})}\right) , \end{aligned}$$

the $$L^2$$ norm,

$$\begin{aligned} {D}_{i,L^2}=\left( \sum _{j=1}^{\tau _i}{\left( w_{ij}{d(t_{ij})}\right) }^2\right) ^{1/2}, \end{aligned}$$

the maximum,

$$\begin{aligned} D_{i,\max }=\sup _{j\in \{1,\dots ,\tau _i\}}\left( {w_{ij}d(t_{ij})}\right) , \end{aligned}$$

and the minimum,

$$\begin{aligned} D_{i,\min }=\min _{j\in \{1,\dots ,\tau _i\}}\left( {w_{ij}d(t_{ij})}\right) , \end{aligned}$$

where $w_{ij}$ is a weighting vector for the combinations across the time course. Non-uniform weightings may be of use if it is desirable to better fit particular parts of the time course, as is common in inference. For example, the steady state may be given greater weighting than the rest of the time series, or there may be some behaviour that occurs at a particular time that is important. In this study, we have used uniform weightings of $w_{ij} = 1$ for all *i* and *j*. An example of the mean and maximum experimental distances are illustrated in Fig. 5.

The range of values for the experimental distances depends on the choice of metric for the point distance and the combination across the experimental time courses. For the Kolmogorov–Smirnov point distance, the theoretical ranges of the experiment distances above are all between zero and one. For the integrated point distances, such as the Wasserstein-1, the experimental distance for this study has a range of zero to approximately 1.5.

### Combined Distance Across Multiple Experiments

For systems with multiple experimental protocols constraining the model, the experimental distances can be used to define a combined distance, $\Delta$. Here we take the combined distance to be the $L^2$-norm or Euclidean combination of the *E* experimental distances,

$$\begin{aligned} \Delta =\left( \sum _{i=1}^EW_iD_i^2\right) ^{1/2}, \end{aligned}$$

where $W_i$ is the weighting of experiment *i* and $D_i$ is the experimental distance of experiment *i*. Note that the particular underlying point and experimental distance choices should be the same across the different experiments in the combination. Again, as with the point and experimental distances, different weightings can be applied to the different experiments, and different definitions for the combination could be considered. We have used uniform weighting with $W_i = 1$ for all *i* in this study.

The range of values for the combined distance again depends on the choice of the underlying point and experiment distances. The theoretical minimum of zero is in practice never achieved due to the limited samples of the data, although the effects of the number of samples of the model can be minimised. Noise in the experimental data will also increase the minimum distance values observed.

## Results

Here we focus on the Kolmogorov–Smirnov and Wasserstein-1 point distances as they are representative of the results for the discrete and integrated point distance classes, respectively. Similar distance landscapes (changes in distance across parameter space) were observed for the other distances in each class; see also Appendix B.

**Fig. 6:**
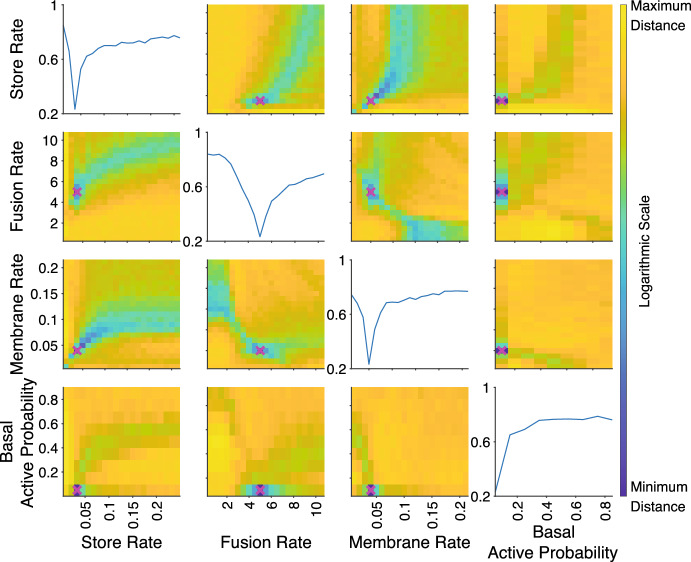
Kolmogorov–Smirnov experiment distance as a function of the system parameters for the transition experiment with synthetic data from parameter set 1. Each subplot varies two model parameters with all other parameters fixed to the set 1 values. The synthetic data parameter values are marked by a magenta cross on each subplot. The colour bar represents the combined distance on a log scale. The diagonal plots show the distance as a function of a single parameter with all other parameters fixed at the synthetic data values (Color figure online)

**Fig. 7:**
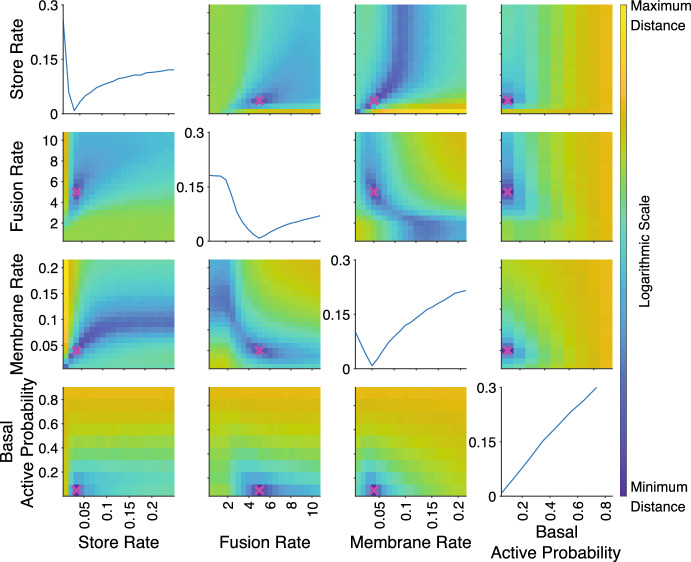
Wasserstein-1 experiment distance as a function of the system parameters for the transition experiment with synthetic data from parameter set 1. Each subplot varies two model parameters with all other parameters fixed to the set 1 values. The synthetic data parameter values are marked by a magenta cross on each subplot. The colour bar represents the experiment distance on a log scale. The diagonal plots show the distance as a function of a single parameter with all other parameters fixed at the synthetic data values (Color figure online)

For the experiment distance, we present the mean of the point distances, ${\bar{D}}_i$, across the time courses. As can be seen in Figs. 6 and 7, this choice of experiment distance resulted in continuous distance values as a function of the parameter values of the model, with the experimental distances derived from Kolmogorov–Smirnov point measures resulting in more abrupt changes as a function of the parameters compared those with underlying Wasserstein-1 point measures. Experimental distances determined using the supremum, $D_{i,max}$, and the minimum, $D_{i,min}$, led to abrupt transitions and discontinuous distances as functions of the parameter values, regardless of point distance measure (not shown). These behaviours are undesirable in the context of this study as we seek a smooth, continuous distance decreasing towards the minimum value to ultimately drive parameter inference. Whilst neither the supremum or minimum could achieve this, the mean remains a viable option to determine the experiment distance.

Varying parameters pairwise about the true values, the landscape of the distances for each of the three experiments in our test study was investigated to determine the existence and location of local minima around the true parameter values. The results for using the mean experimental distance, $\bar{D_i}$, for the transition experiment of synthetic data set 1 are presented as an example in Figs. 6 and 7, using the Kolmogorov–Smirnov and the Wasserstein-1 point distances respectively. It can be seen in each case that the experiment distance has a minimum (but non-zero) value at the true parameter values but there is significant difference in the sensitivities of the two underlying classes of point distances. It can be seen that for the Wasserstein-1 experiment distance there is a more gradual descent to the minimum compared to those in the Kolmogorov–Smirnov experiment distance. Similar results were obtained for the basal and insulin uptake experiments. Similar results were observed for the experiment distances using other point measures, a sample of which are shown in Appendix B, Fig. 13, with the integrated point measures resulting in more gradual transitions to the minimum and the discrete point measures having abrupt transitions as the parameters approach the true values. Note that in all cases the true parameter values of the synthetic data coincided with the minimum in the experiment distances.

**Fig. 8:**
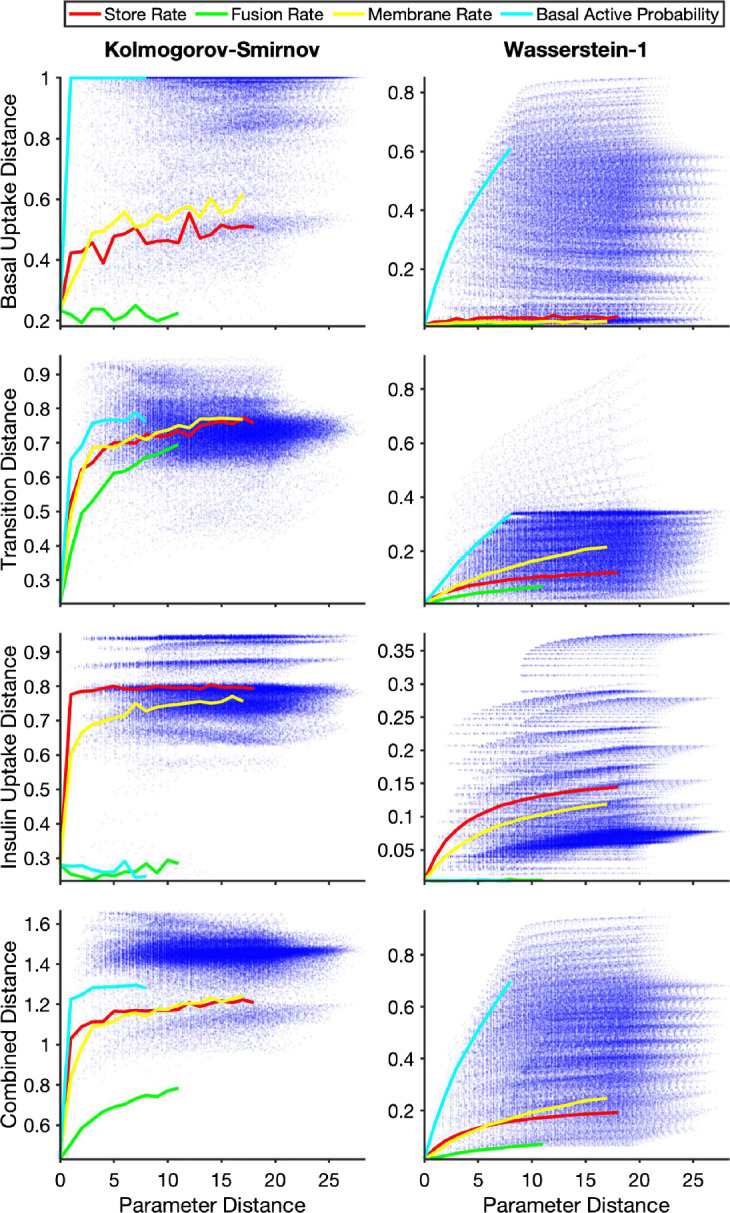
Experiment and combined distances as a function of the parameter distance. Each blue dot represents the results for a particular point in parameter space. The red, green, yellow and cyan lines indicate points that change only the store rate, fusion rate, membrane rate and basal active probability respectively, with the other parameters set at the true parameter values. The rows show the experiment (Basal Uptake, Transition, and Insulin Uptake) and combined distance measures, with the distances based on the Kolmogorov–Smirnov point measure in the left column, and those based on the Wasserstein-1 point measure in the right column. The experiment distance was determined as the mean of the point measures, and the combined distance was the $L^2$-norm of the three experiment distances (Color figure online)

To visualise the distance landscape in higher dimensions we calculated the combined distances across the three experiments for each point in parameter space and mapped them as a function of the parameter distance (defined in Sect. 2.4). Figure 8 shows the combined distance determined using the Kolmogorov–Smirnov and Wasserstein-1 point and mean experimental distances as a function of the parameter distance for synthetic data set 1. Similar results were obtained for the other synthetic data sets and for different combinations of distance metrics (see Appendix B, Fig. 15). Each blue point in the figures represents a particular parameter combination. The solid curves show the trend of the combined distance as a single parameter is varied while the other parameters are held at the synthetic data values.

It can be seen that both the experimental and combined distances decrease as the parameters approach the true values. When the Kolmogorov–Smirnov and other discrete point measures are used, the distances remain relatively large until close to the true parameter values, with a rapid decrease to the minimum at the true parameter values. The distances based on the Wasserstein-1 and other integrated point measures, on the other hand, show smooth decreases as the parameters approach the true values.

It can also be seen that the distance is more sensitive to changes in some parameters than others, and that this is more visible in the distances employing the Wasserstein-1 point measure. For example, in the basal uptake experiment in Fig. 8, changes in the basal active probability have the largest effect on the distance—it has the highest single parameter sensitivity—and the distance smoothly decreases as the true parameter value is approached. The corresponding distances based on the Kolmogorov–Smirnov point measure also show that this is the most sensitive parameter, but the approach to the minimum value abruptly switches from the maximum to minimum values as the true parameter values are approached. Exploring the distance landscape for the different experiments, it can be seen that the relative sensitivities of parameters differ between the experiments, e.g., the membrane rate is more sensitive than the store rate in the transition experiment, but this relation is switched for the insulin uptake experiment. This illustrates that the visualisation of the single parameter sensitivities for experimental distances as a function of the parameter distance may be a useful approach when undertaking parameter inference and model selection.

**Fig. 9:**
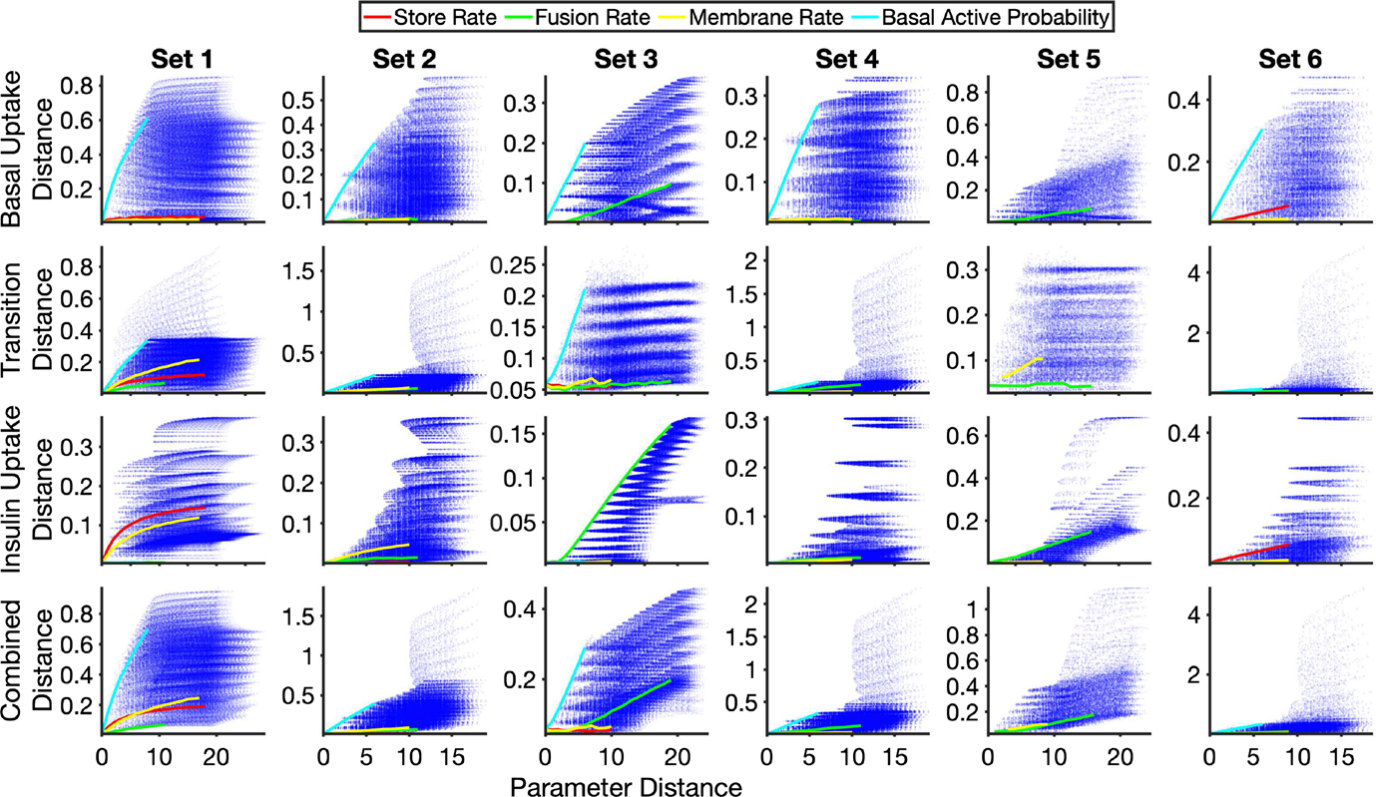
Combined distance as a function of parameter distance for six different synthetic data sets. Each blue dot represents the results for a particular point in parameter space. The red, green, yellow and cyan lines indicate points that change only the store rate, fusion rate, membrane rate and basal active probability respectively, with the other parameters set at the true parameter values. The rows show the experiment (Basal Uptake, Transition, and Insulin Uptake) and combined distance measures, with the distances based on the Wasserstein-1 point measure, the mean experiment distance and the $L^2$-norm for the combined distances (Color figure online)

The behaviour of the system, and the effects of single parameter perturbations can depend on the region of parameter space in which the true values lie. For this system, however, similar trends for the distances as functions of the parameter distance and the parameter sensitivities were observed for the different synthetic data sets. Using the Wasserstein-1 point measure, the (mean) experiment and combined distances as a function of the parameter distance for each synthetic data set is shown in Fig. 9. Each set shows the general trend of distances decreasing as parameter distance decreases.

Effects of adding noise to the synthetic data As the model is stochastic, the minimum distance that can be achieved is non-zero, even when the model is evaluated using the same parameters as the synthetic data; i.e., drawing the exact same sample points is unlikely. This limits the depth of minimums that can be found in the distance landscape. There is also an upper bound that exists for the distance between datasets, i.e., there exists a limit to quantitatively distinguishing between two dissimilar models.

**Fig. 10:**
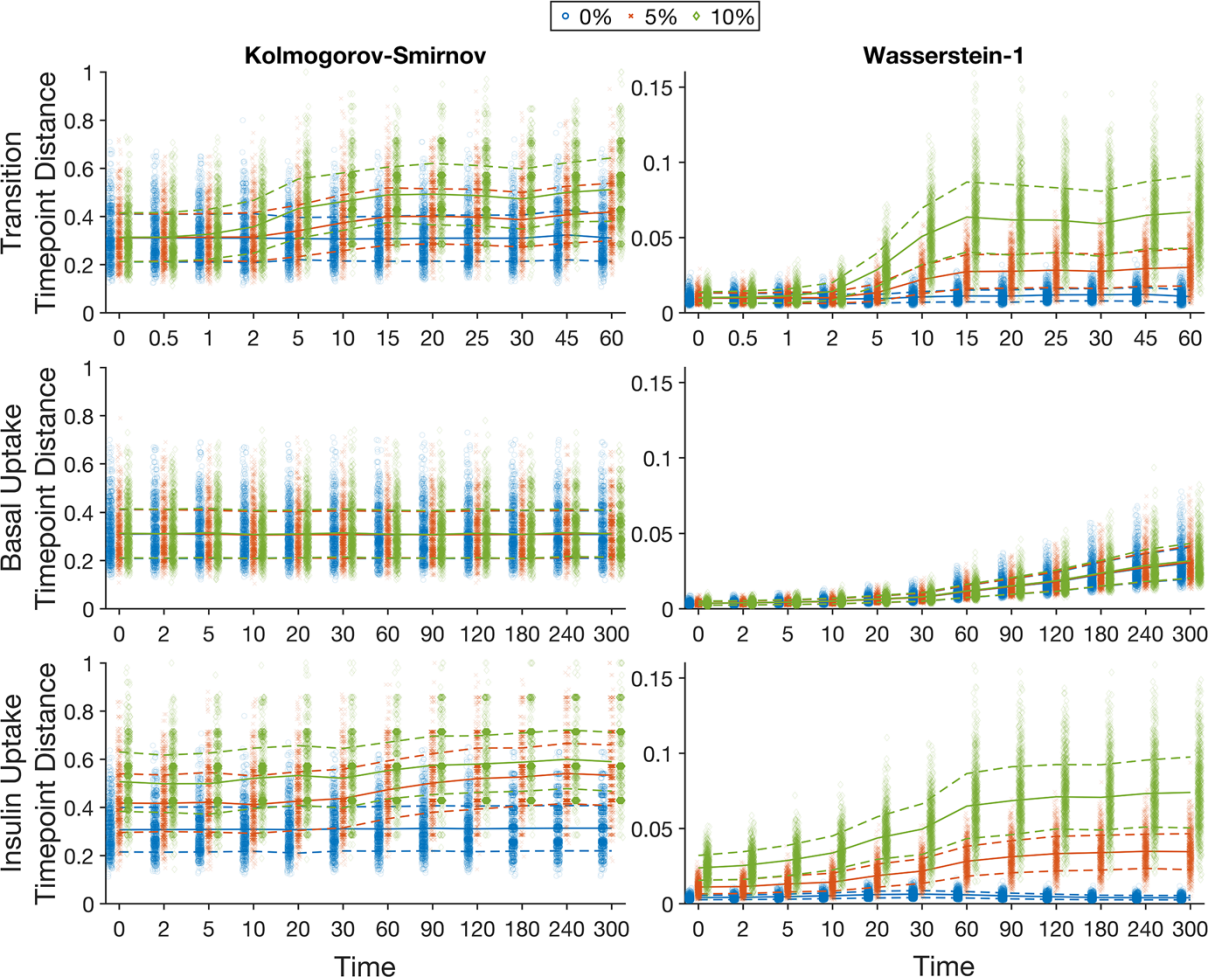
Point distances across the time courses of the three experiments for synthetic data for parameter set 1. At each time point the distance was calculated for 1000 re-samples of the seven synthetic data points using the Kolmogorov–Smirnov and Wasserstein-1 point distances. The point distances were then calculated for the same synthetic data sets with additive normal noise of 0%, 5% and 10%. The means and standard deviations are represented by solid and dashed lines respectively. Note the differing scales on each subplot and the categorical clustering of the results at each time point (Color figure online)

To explore the practical ranges of the distances and the effects of experimental noise for the current scenario each synthetic data set was re-created by re-sampling the model outputs 1000 times (following the methodology outlined in Sect. 2.4). Normal (Gaussian distributed) noise at different levels was then added to each of these synthetic data sets and the point distances determined for each of the experimental time courses, Fig. 10. It can be seen that the variance of the Wasserstein-1 point distances due to re-sampling the synthetic data is lower than the Kolmogorov–Smirnov distance at all points in the time courses, both with and without noise. Indeed, the Kolmogorov–Smirnov distances for the re-sampled synthetic data without noise includes values close to one, the theoretical maximum value.

**Fig. 11:**
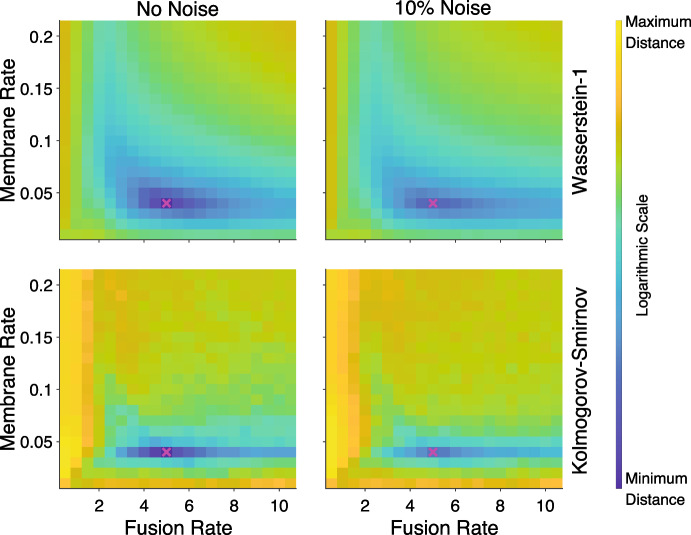
Combined distance, mean across time and $L^2$-norm across experiments, as a function of the fusion rate and membrane rate for the transition experiment with synthetic data from parameter set 1 with no noise (left), and with the addition of 10% Gaussian noise (right). The combined distance is shown using the Wasserstein-1 point measure (top row) and the Kolmogorov–Smirnov point measure (bottom row). Each subplot varies the fusion rate and membrane rate, on the horizontal and vertical axes respectively, with all other parameters fixed to the parameter set 1 values. The synthetic data parameter values are marked by a magenta cross on each subplot. The colour bar represents the combined distance on a log scale, set to the minimum and maximum of the two different distances with no noise (Note the actual values for the two distances are different in magnitude) (Color figure online)

Although the variance of the distances using the Wasserstein-1 point measure is relatively small, the mean combined distance increases with noise, effectively increasing the height of the local minima in the landscape. The impacts noise has on the combined distance landscape for varying parameter values, with and without noise for synthetic data set 1 is illustrated in Fig. 11. For the distances using the Kolmogorov–Smirnov point measure there is still a clear crevasse containing the minimum in the case of noisy data, even though it is less well-defined than with zero noise. It is important to note however that the observed minima for the noisy synthetic data move away from the true parameter values. With the addition of noise to the data the minima of the distances using the Wasserstein-1 point measures become shallower and are not as pronounced compared to surrounding points, but the landscape still provides a smooth descent towards the true minima.

**Fig. 12:**
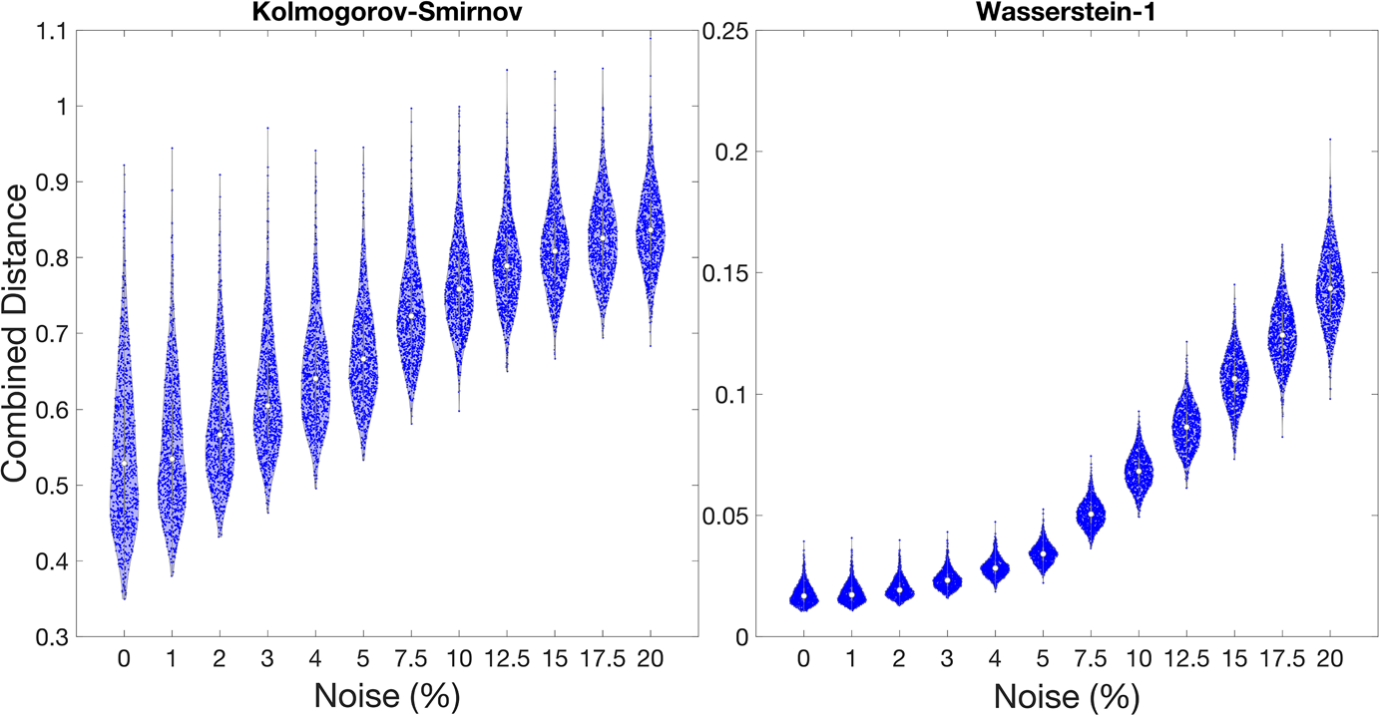
Combined distances for synthetic data set 1. The points indicate the distances for each of the 1000 re-samples of the seven synthetic data points with normal noise up to 20% added. The combined distances were determined using the $L^2$ normal of the three mean experiment distances and the Kolmogorov–Smirnov (left) and Wasserstein-1 (right) point distances. Note differing vertical scales (Color figure online)

The effects of the addition of noise on the mean experiment and $L^2$ combined distances are shown in Appendix B, Fig. 12. Similar trends for increasing noise were observed for each synthetic data set investigated, as shown in Fig. 16. The use of the Wasserstein-1 point distance causes smaller variations due to re-sampling than when the Kolmogorov–Smirnov point measure is used. The variance of the Kolmogorov–Smirnov combined distance is relatively insensitive to additional noise, but starts from a high base—there is large variation even with zero noise. Although the variance and mean of the Wasserstein-1 combined distance increases with noise, it remains much more clustered at 20% noise than the Kolmogorov–Smirnov combined distance at zero noise.

The investigation above was repeated with the addition of noise drawn from a uniform distribution to see whether the trends were dependent upon the choice of noise, Appendix B, Fig. 17. Note, the choice of noise distribution had minimal impact on the observations using the Kolmogorov–Smirnov point distance. However, for those using the Wasserstein-1 point distance, the addition of uniform noise did not increase the distances or variance as much as the addition of normally distributed noise. From here, we discuss only the case of adding normal noise.

## Discussion

Synthetic data sets, with known parameter values, were generated from the queuing model. These synthetic data sets were chosen to provide good qualitative representations of the experimental data. The model was then simulated for a variety of parameter combinations and distances from the output to the synthetic data sets were calculated. Distance landscapes around the true parameter values were created to investigate whether the distances had appropriate characteristics to assess the model and parameter selection.

For a distance measure to be useful for parameter inference, the minimum distance in the parameter range needs to occur (ideally) at the true parameter values, or, in a basin near the true parameter values. For systems constrained by multiple experimental data sets (controlled by the same underlying parameters), each of the distance landscapes would be expected to converge to minima at the true parameter values. Similarly, we expect the minimum combined distance to occur at the true parameter values. For this minimum to be identified by a fitting algorithm, the landscape of the combined distance in parameter space should have some directed and, ideally, smooth descent to the minimum.

To determine a suitable distance comparator for inference, we proposed a three-level approach: a point distance across each experimental time course, an experiment distance aggregating the point distances, and a combined distance taking all the experiments into account.

The point distances are based on the ECDFs of the data and model outputs at each point of the time courses of each experiment. Here the experimental data was sparse, with only seven samples at each time point of each experiment. It was found that the resulting distances were robust to sampling level of the model output if more than 100 samples were used. In this study, two classes of point measures were considered: discrete point measures such as the Kolmogorov–Smirnov metric and integrated point measures such as the Wasserstein-1 distance. The integrated point measures were found to produce a smoother continuum of values compared to the discrete point measures, see Fig. 5, mainly due to the tendency of the discrete measures such as the Kolmogorov–Smirnov to have bimodal outputs: maximum values for large parts of the parameter space with other values observed only observed at parameters very close to the true values.

The distances at each point were then aggregated to an experiment distance over the time course. For the data and model explored in this study, it was found that experiment distances aggregated by taking the mean of the point distances over the time course with uniform weighting resulted in distances that decreased as a function of parameter distance, Fig. 8. However, other experiment distance aggregations may also be suitable, and the choice should be explored when applying the algorithm to different systems. In particular, knowledge of the structure of the data may inform a suitable weighting for the aggregation. For example, if it is known that an experimental setup is less reliable at early time points, one may wish to weight these points less than other time points. In the case of combinations of data from multiple sources, the weighting can reflect the relative veracity and reliability of the data sources.

**Fig. 13:**
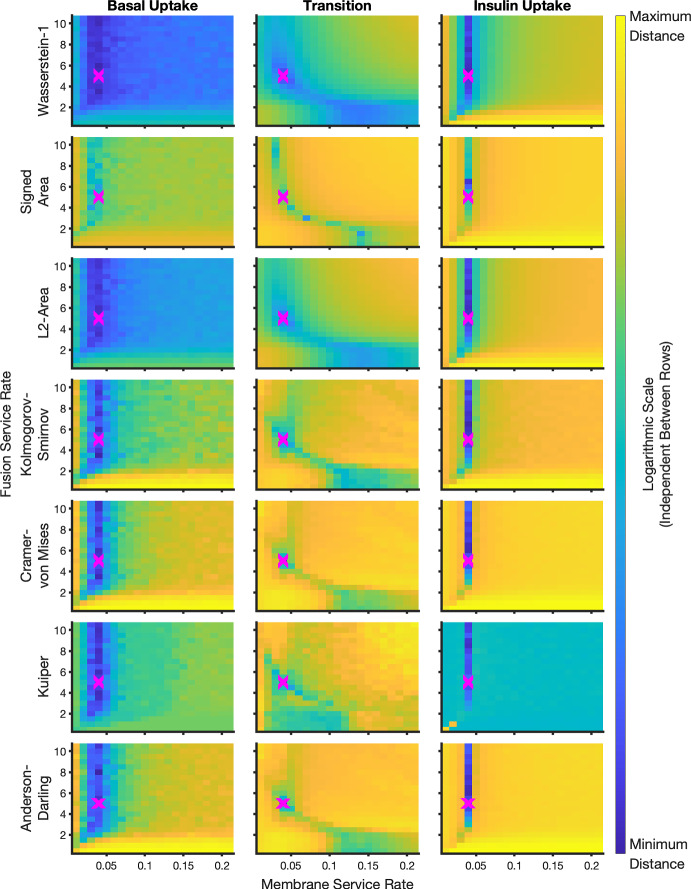
Experiment distance as a function of parameter values. The experiments are shown in the columns and the distances using different point measures in the rows. Each subplot varies the membrane rate and the store rate with all other parameters set at the synthetic data set 1 values. The colour scale shows the minimum and maximum distance seen in the parameter space for that distance measure (note these are different values for the different distances) on a log scale. The true parameter values are marked with a red cross (Color figure online)

**Fig. 15:**
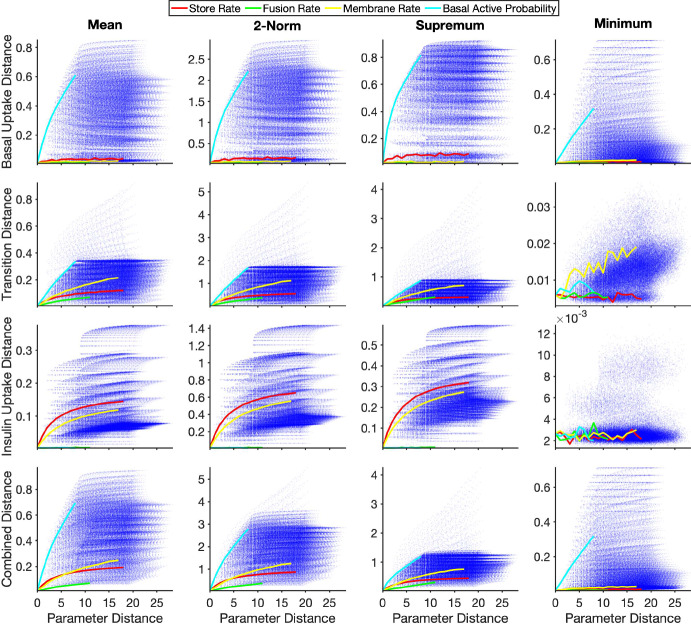
Combined distances for the six synthetic data sets. The points indicate the distances for each of the 1000 re-samples of the seven synthetic data points. The combined distances were determined using the $L^2$ normal of the three mean experiment distances for the indicated point measures. The distance measures cluster based on their classification as a point distance or integrated distance. The exception to this is the Cramer–von Mises which behaves more similarly to the integrated distances as it takes all of the samples in the ECDF into account (Color figure online)

**Fig. 16:**
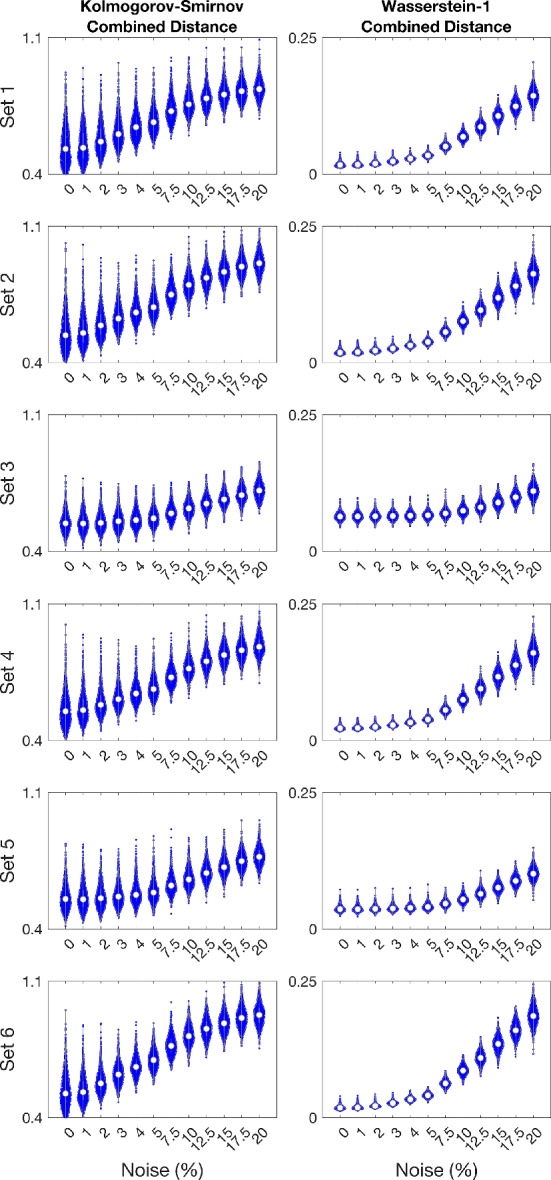
Distance as a function of parameter distance for synthetic data set 1. Each blue dot represents the results for a particular point in parameter space. The red, green, yellow and cyan lines indicate points that change only the store rate, fusion rate, membrane rate and basal active probability respectively, with the other parameters set at the true parameter values. The rows show the experiment (Basal Uptake, Transition, and Insulin Uptake) and combined distance measures, with the distances based on the Wasserstein-1 point measure. The columns show the effect of different aggregations for the experiment distance. The combined distance was the $L^2$-norm of the experimental distances (Color figure online)

**Fig. 17:**
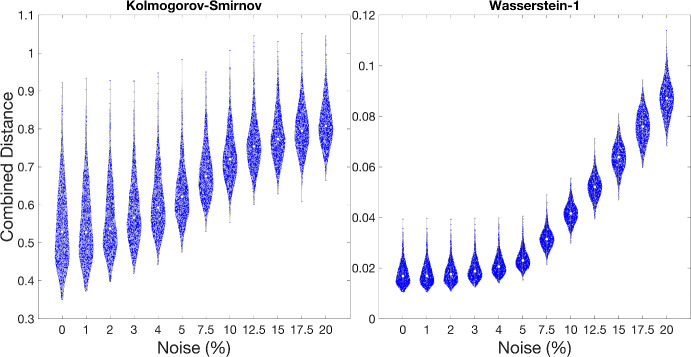
Combined distances for synthetic data set 1. The points indicates the distances for each of the 1000 re-samples of the seven synthetic data points with uniform noise up to 20% added. The combined distances were determined using the $L^2$ norm of the three mean experiment distances and the Kolmogorov–Smirnov (left) and Wasserstein-1 (right) point distances. Note differing vertical scales. Note the similarity to Fig. 12 which showed the effect of additive normal noise. In this case however the scale of the Wasserstein-1 distances is smaller, i.e., adding uniform noise does not increase the distance as much as the addition of normal noise, although the results for the Kolmogorov–Smirnov point distances are similar (Color figure online)

The experiment and combined distances inherit the characteristics of the underlying point measure. The experiment distances using the Wasserstein-1 point measures are smoother functions of the parameter values than those using the Kolmogorov–Smirnov point measures, Fig. 8. Experiment distances using the Kolmogorov–Smirnov or other discrete point measures are in general only sensitive to parameter changes close to the true parameter values. Away from the true parameters, these distances plateau to a small range of values. If the approximate values of the parameters are unknown this large plateau can make localisation of the region containing the minimum difficult, and so these distances are not appropriate for parameter optimisation based on gradient methods. However, the distances employing the Wasserstein-1 or other integrated point measures are sensitive across a larger range of parameter space surrounding the minima. Thus, these distances are appropriate for directed parameter inference, and remove the need for a ‘good’ start point for the search. The sensitivity of the parameters may be different for different experiments, Appendix B, Fig. 13, so a visualisation of the experiment distance as a function of the parameters is suggested when the algorithm is applied to a new system.

The overall combined distance was determined as the $L^2$-norm, Appendix B, Fig. 13, again inheriting the characteristics of the underlying point and experiment distances. It can be seen that both the discrete and integrated point measures result in a combined distance that decreases as the parameters approach their true values, albeit more abruptly in the case of the discrete point measures.

Robustness of the measure to added noise When using real experimental data (especially data with small sample sizes) it is unknown to what degree the sample is truly representative of the system. Moreover, it is sometimes difficult to control the level of errors present in the data due to, for example, limitations in the precision of measuring equipment, or approximations made in reported values. A distance that can detect the true parameter values, even when the sample may not be truly representative of a distribution, is thus beneficial.

Investigations re-sampling the synthetic data resulted in a distribution of distances even when the model was simulated using the true parameter values, Figs. 10, 11 and 12. Noise tends to reduce the depth of the minima and makes them harder to detect. For the discrete point measures this may mean that the minima cannot be detected with confidence as the depth of the minima was similar in magnitude to the variability of the distances. This is not due to the sampling of the model output, which we can control—it was found that using 100 or more samples of the model output resulted in reproducible distances for a given data set, but rather due to the variability of the sparse experimental data. Due to the bimodal nature of the outputs of the Kolmogorov–Smirnov measure, the distances based on this measure in the re-sampling investigation were close to the maximum possible value, even without the addition of noise, Fig. 12. The Wasserstein-1 distance was found to have a smaller variance than the Kolmogorov–Smirnov distance when re-sampling the experimental data, including when noise was added. This suggests that the Wasserstein-1 distance is more robust to the particular experimental samples and experimental noise in the data: although the minima in the distance landscape was found to be less pronounced with increased noise, a minima still exists for moderate levels of noise (10%). This means the correct region of parameter space could still be found, albeit with less confidence in the found values. The Wasserstein-1 distance remains viable even with 10% added noise, and experimental data with suspected noise higher than this is unlikely to be robust.

Distances based on the Wasserstein-1 measure, representative of the other measures of the integrated point distance class, are sensitive to changes in parameters over a large region of parameter space, and approach the minimum smoothly as a single parameter is varied. They are also robust under additional noise in the data. Using this distance, a directed inference of the system is possible. For some data samples, the distances based on the Kolmogorov–Smirnov and other discrete point measures can provide a highly localised minimum, and could be used to check and refine parameter values found by the Wasserstein-1 distance. However, for a ‘bad’ sample of the data (whether from the particular sampling or noise) we have observed that the Kolmogorov–Smirnov distance may fail to localise the minimum and hence for the small sample sizes considered here, the Kolmogorov–Smirnov is too variable for practical use. However, if more data were available at each time point, the variance may be better constrained.

This study demonstrates the distance measures for a specific model and data set, motivated by a biological system of interest to the authors. The comparison between integrated and discrete based measures undertaken in this case suggests integrated measures are more capable of smoothing descents to a minimum for other similar stochastic models. The discrete point distances are highly effective classifiers of pairs of distributions, i.e., they can determine if two samples are from the same distribution or not. However, this classification is largely binary (despite the distance being able to take a continuum of values). Thus when model fitting, especially with a computationally expensive model, the binary nature of these measures mean finding the region(s) of parameter space where the model and experiments match can be problematic. The integrated distances here provide a smoother and more continuous notion of ‘closeness’ between the model and experiments to better effect parameter inference.

A purposeful limitation of our test of the distance quantification was to limit the observability of the system and thus limit the amount the system could be measured. Systems with greater observability, i.e., able to measure their system at more than a single location, may find a greater range of alternative distances suitable to describe the correspondence of the model outputs and the observations. Indeed, if a high number of data points are available many time point distances become available, such as distribution density fitting and moment matching. Here, we sought to address quantifying the distance in the context of sparse data, which is a common problem for many studies in the biological sciences.

This work suggests that in employing this algorithmic approach for the distance for other systems the integrated class of point distances are a good starting point, especially if the parameter space is not well constrained. In this paper synthetic data and an extensive parameter space was employed to fully test the efficacy of the different distance measures. It is yet to be seen if the best choices of distances found here apply to all stochastic systems without prior investigations with synthetic data, as undertaken here. However, we believe such an extensive testing of the distance with synthetic data, is not necessary before each implementation as it becomes obvious from smaller scale tests with the actual data whether the particular distance chosen in the algorithm smoothly decreases to minima in the parameter space.

## Conclusion

We have identified a hierarchical approach to quantitatively compare stochastic models to stochastic data using an algorithm to combine point distances, aggregate to individual experiment distances and create a combined distance across experiments. We found that the choice of point distance is vital, with the choice of aggregation mechanism having less effect on the final result. The distances using the integrated point metrics were found to be smoothly continuous functions of the parameter distance as well as robust to noise and the variability of the underlying data.

The distances based on the integrated point measures form a robust basis to compare the stochastic model to the data, appropriate for parameter inference. The algorithmic approach presented here provides a mechanism to compare stochastic models to stochastic data, quantitatively taking into account the variability in both systems.

## Figures and Tables

**Fig. 14:**
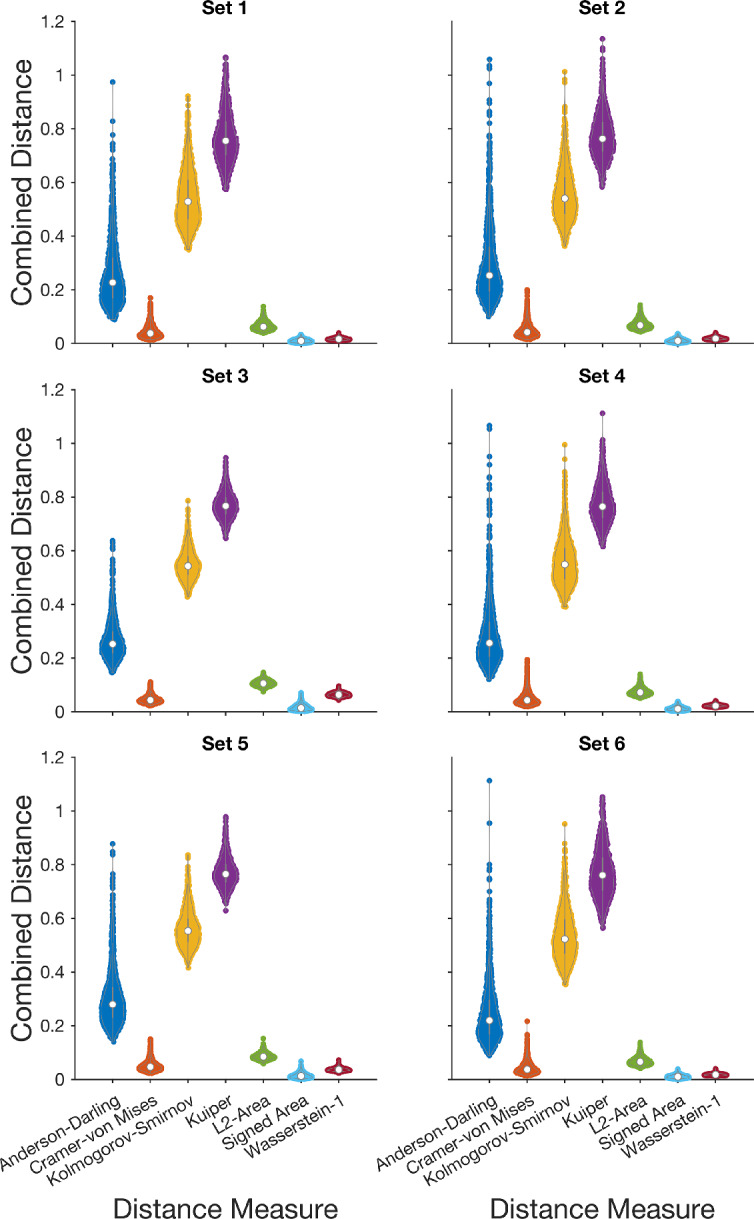
Combined distances for each synthetic data set. The points indicate the distances for each of the 1000 re-samples of the seven synthetic data points with normal noise up to 20% added. The combined distances were determined using the $L^2$ normal of the three mean experiment distances and the Kolmogorov–Smirnov (left column) and Wasserstein-1 (right column) point distances. Note differing vertical scales between columns. Similar trends are seen across each parameter set. Noting that high levels of noise increase the distance more in some parameter sets (Color figure online)

## Appendix A: Supplementary Distance Measures

### Appendix A.1: Example Discrete Point Distances

Kuiper Distance Kuiper’s distance (Kuiper 1960) is closely related to the Kolmogorov–Smirnov distance but rather than only considering the supremum, the infimum is also taken. The distance is defined as

4 $$\begin{aligned} d_K(t_{ij}) = \sup _x (F(x,t_{ij}) - G(x,t_{ij})) - \inf _x (F(x,t_{ij}) - G(x,t_{ij})). \end{aligned}$$

The original intention of the Kuiper test was to extend the Kolmogorov–Smirnov test to be applicable to points on a circle rather than on a line. Here, the distance is used to consider a greater portion of the ECDF values than the Kolmogorov–Smirnov distance. The Kolmogorov–Smirnov distance is more sensitive around the median of the data whereas the Kuiper distance is equally sensitive over the entire distribution (Lanzante 2021).

Note the definition in Eq. (4) is not favourable if the two ECDFs do not intersect, i.e. if $\inf _x (F(x,t_i) - G(x,t_i)$ is positive. For use as a distance the definition given in (Lanzante 2021) will be used

$$\begin{aligned} d_K(t_{ij}) = \vert \sup _x (F(x,t_{ij}) - G(x,t_{ij}))\vert + \vert \sup _x (G(x,t_{ij}) - F(x,t_{ij})) \vert . \end{aligned}$$

Advantages of these distances include: they are already used for goodness of fit between one-dimensional probability distributions, they do not assume any family of distribution, and the distance is independent of the number of samples. Independence from sample size is useful when considering multiple experiments that may have different sample sizes—it eliminates the need to weight experiments based on sample size.

These distances suffer from a lack of sensitivity when the range of samples from the two sets do not overlap. If there is no overlap in the range of samples then $d_{KS} = 1 = d_K$, which gives no information on ‘closeness’ of the distributions until they begin to overlap. Which is not useful when the aim is inference.

The Kolmogorov–Smirnov distance and the Kuiper distance are distances between samples based on the maximum difference in ECDF values. These distances are well-known and are often used as two-sample tests. They effectively characterise whether two samples are from the same distribution or not. However, for the purpose of inference, and needing sensitivity across the parameter space, they can be limited as they do not take into account all values. And, in the case of the Kolmogorov–Smirnov distance, have a maximum distance of one. This maximum distance occurs when the samples are disjoint, i.e., there is no overlap in the sample ranges. This indicates that the distance would not be sensitive to parameter changes when the samples are very far apart.

The following distances, the Cramer–von Mises and Anderson Darling distances, use all data samples to determine a distance between two samples. By taking into account all sample points they may be less sensitive to outliers in the data than the Kolmogorov–Smirnov and Kuiper distances.

A Distance of the Cramer–von Mises Type The Cramer-von Mises criterion (Cramér 1928) is defined as

$$\begin{aligned} \omega ^2 = \int _{-\infty }^\infty \left[ F_N(x) - F(x)\right] ^2 \,dF(x), \end{aligned}$$

where $F_N(x)$ is the ECDF of the sample and *F*(*x*) is a specified continuous distribution. The criterion is used for testing the sample from $F_N(x)$ has been drawn from the distribution *F*(*x*). A two sample analogue of the distance, given in Anderson (1962), is defined by the Stieltjes integral

5 $$\begin{aligned} d_{CVM}(t_{ij}) = \int _{-\infty }^\infty \left[ F_{L_{ij}}(x,t_{ij}) - G_{R_{ij}}(x,t_{ij})\right] ^2 \,dH_{L_{ij}+R_{ij}}(x), \end{aligned}$$

where $H_{L_{ij}+R_{ij}}(x)$ is the ECDF of the combined sample. Note, the test statistic includes a factor $\frac{L_{ij}R_{ij}}{L_{ij}+R_{ij}}$ in front of the integral, in this investigation it has been omitted as it scales the integral similarly for all test cases. The Cramer–von Mises distance is a quadratic distance and considers equally all data points. It is analogous to a least squares (sum of squares) distance by considering the square distance between each data point in the ECDF.

Solving the Stieltjes integral in the usual way gives

6 $$\begin{aligned} d_{CVM}(t_{ij})&= \sum _{n=1}^{L_{ij}+R_{ij}-1}\left[ F_{L_{ij}}(x_{n},t_{ij}) - G_{R_{ij}}(x_{n},t_{ij})\right] ^2\nonumber \\&\quad \left( dH_{L_{ij}+R_{ij}}(x_{n+1},t_{ij})-dH_{L_{ij}+R_{ij}}(x_n,t_{ij})\right) , \end{aligned}$$

7 $$\begin{aligned}&= \frac{1}{L_{ij}+R_{ij}} \sum _{n=1}^{L_{ij}+R_{ij}-1}\left[ F_{L_{ij}}(x_{n},t_{ij}) - G_{R_{ij}}(x_{n},t_{ij})\right] ^2. \end{aligned}$$

Note, the summation to $L_{ij}+R_{ij}-1$ instead of $L_{ij}+R_{ij}$ allows for the Stieltjes integral to be resolved but does not impact the final sum by missing the square difference of the $L_{ij}+R_{ij}$th point as $F_{L_{ij}}(x_{L_{ij}+R_{ij}},t_i) - G_{R_{ij}}(x_{L_{ij}+R_{ij}},t_i) = 0$ by the definition of the ECDF. Although classified here as a discrete point distance, the Cramer–von Mises distance displays behaviour more similar to that of the integrated point distances (see Fig. 14). This is likely a result of this distance measure using every sample point to determine a distance, rather than only select samples like the Kolmogorov–Smirnov or Anderson–Darling distances.

A Distance of the Anderson–Darling Type The Anderson–Darling test (Anderson and Darling 1952) is similar to the Cramer–von Mises but chooses a weight function to weight the tails of the distribution. The two sample Anderson–Darling distance (Pettitt 1976) is the Stieltjes integral

$$\begin{aligned} d_{AD}(t_{ij}) = \int _{-\infty }^\infty \frac{\left[ F_{L_{ij}}(x,t_i) - G_{R_{ij}}(x,t_{ij})\right] ^2}{H_{N+M}(x,t_{ij}) (1-H_{L_{ij}+R_{ij}}(x,t_{ij}))} \,dH_{L_{ij}+R_{ij}}(x,t_{ij}). \end{aligned}$$

Solving the integral gives the summation

$$\begin{aligned} d_{AD}(t_{ij}) = \frac{1}{L_{ij}+R_{ij}} \sum _{n=1}^{L_{ij}+R_{ij}-1}\frac{\left[ F_{L_{ij}}(x_{n},t_{ij}) - G_{R_{ij}}(x_{n},t_{ij})\right] ^2}{{H_{L_{ij}+R_{ij}} (x_n,t_{ij})(1-H_{L_{ij}+R_{ij}}(x_n,t_{ij}))}}. \end{aligned}$$

These distances are both common in testing if two samples are from the same distribution. In this study, we investigate if they are sensitive to deviations in parameters in a way that could drive inference when multiple distributions must be compared at once. These distances improve on the Kolmogorov–Smirnov and Kuiper distances by using all samples in the distance calculation, which allows for a greater range of distances across parameter space.

### Appendix A.2: Integrated Point Distances

Signed Area We consider an integrated distance, the signed area between the two ECDFs,

$$\begin{aligned} d_{SA}(t_{ij}) = \int _{-\infty }^\infty F(x,t_{ij}) - G(x,t_{ij})\,dx. \end{aligned}$$

This formulation allows for negative distances. So, if the ECDFs intersect some portion of the area enclosed by the ECDFs will subtract from the overall distance.

L2-Area We define a ‘squared’ L2-area as

8 $$\begin{aligned} d_{L2A}(t_{ij}) = \left( \int _{-\infty }^\infty \vert F(x,t_{ij}) - G(x,t_{ij})\vert ^2 \,dx\right) ^{1/2}. \end{aligned}$$

This distance takes inspiration from the idea of a least-squares type distance and the Wasserstein distances, in that it is an integrated distance but considers squaring the vertical distance between the ECDFs when calculating the area between the ECDFs.

## Appendix B: Supplementary Table and Figures

Table 1 shows the parameter sets used to create the synthetic data sets as well as the range of parameter values used in this study. Figures 13 and 14 show the distance landscapes for all distance measures considered in the study. Figure 15 shows distance as a function of parameter space for each of the synthetic data sets investigated for different combinations of the experiment distance. Figure 16 shows the effect on the combined distance from the addition of Gaussian noise to each of the six synthetic parameter sets investigated. Figure 17 shows the effects of uniform noise (in place of Gaussian noise) on the distance.

**Table 1:** Parameter values used to create synthetic data sets in this study

	Set 1	Set 2	Set 3	Set 4	Set 5	Set 6
Endosome store service rate—*the reciprocal of the time spent in the endosome store plus the time to exit the store and arrive at a microtubule*						
$\mu _S$	$\frac{1}{30}$	0.12875	0.12875	0.12875	0.37625	0.12875
Range	[0.01,0.2433]	[0.005, 0.2525]	[0.005, 0.2525]	[0.005, 0.2525]	[0.2525, 0.5]	[0.005, 0.2525]
Microtubule service rate—*the reciprocal of the time taken for a vesicle to transit the length of a microtubule if unimpeded*						
$\mu _M$	0.3	0.3	0.3	0.3	3	3
Fixed	–	–	–	–	–	–
Fusion site service rate—*the reciprocal of the time required for a fusion site to process and fuse the vesicle to the plasma membrane*						
$\mu _F$	5	5	0.1	5	0.1	20
Range	[0.5, 10.5]	[0.5, 10.5]	[0.05, 1.05]	[0.5, 10.5]	[0.05, 1.05]	[2, 42]
Plasma membrane service rate—*the reciprocal of the time taken for the vesicle to reform and return to the internal endosome store*						
$\mu _P$	0.04	0.12875	0.37625	0.37625	0.12875	0.37625
Range	[0.01, 0.21]	[0.005, 0.2525]	[0.2525, 0.5]	[0.2525, 0.5]	[0.005, 0.2525]	[0.2525, 0.5]
Active probability—*the probability of a fusion site being active*						
Basal state						
$p_B$	0.05	0.3	0.3	0.3	0.3	0.3
Range	[0.05, 0.85]	[0.1, 0.9]	[0.1, 0.9]	[0.1, 0.9]	[0.1, 0.9]	[0.1, 0.9]
Insulin state						
$p_I$	0.9	0.9	0.9	0.9	0.9	0.9
Fixed	–	–	–	–	–	–
Number of customers						
*N*	25,000	25,000	25,000	25,000	25,000	25,000
Fixed	–	–	–	–	–	–
Number of microtubules						
*M*	200	200	200	200	200	200
Fixed	–	–	–	–	–	–
Microtubule capacity						
*L*	50	50	50	50	50	50
Fixed	–	–	–	–	–	–

The basal steady state corresponds to an insulin level of 0 nM and the insulin steady state corresponds to an experimental insulin level of 100 nM. The parameter values for each set are shown, with the range of values used in the study shown in brackets. Note that values for the Microtubule Service Rate, the Active Probability Insulin State, Number of Customers, Number of Microtubules, and Microtubule Capacity were fixed for all simulation experiments
